# Extending the limits of algal growth and stress resilience – the desert alga *Chlorella ohadii* as an emerging model

**DOI:** 10.1111/nph.71587

**Published:** 2026-10-01

**Authors:** Or Geffen, Hilla Matityahu, Iftach Yacoby, Michal Breker, Haim Treves

**Affiliations:** ^1^ Systems Plant Metabolism, Faculty of Biology RPTU 67663 Kaiserslautern Germany; ^2^ Department of Plant and Environmental Sciences, The Alexander Silberman Institute of Life Sciences The Hebrew University of Jerusalem 91904 Jerusalem Israel; ^3^ School of Plant Sciences and Food Security, The George S. Wise Faculty of Life Sciences Tel Aviv University Ramat Aviv 97801 Tel Aviv Israel

**Keywords:** abiotic stress, algae, *Chlorella ohadii*, metabolism, model organisms, photosynthesis

## Abstract

Since its isolation from the sand dunes of the Negev Desert over a decade ago, the unicellular green alga *Chlorella ohadii* has turned into a reference organism for studying photosynthesis, metabolism, and abiotic stress response. Much of *C. ohadii*'s appeal as an emerging model lies in its robust fast growth, comparable to yeast even as a photoautotroph. Hence, the detailed physiological, biochemical, and molecular studies that followed rapidly, together with extensive omics datasets, have revealed several of its intriguing biological features. We provide a historical perspective unfolding the rise of this species from survival in the harsh desert crusts to a widely used standard for photodamage resistance, dynamic metabolism, and elevated growth and photosynthetic performance, frequently with prominent relevance for plant research. We also spotlight how this case study enables exploration of photosynthesis in harsh habitats. Further, we discuss challenges in the development of genetic tools for the growing community studying *C. ohadii*, and current biotechnological approaches aiming to exploit its prime genetic potential. Finally, we refer to guidelines of good practice for *C. ohadii* research as a highly stress‐resistant model.

## Graphical abstract

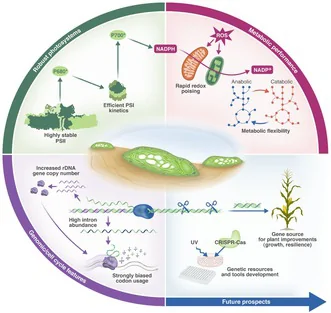

## Introduction – the life history of *Chlorella ohadii*

Abiotic stress response is a major driver of acclimation and adaptation processes in land plants, due to their sessile nature. Throughout many decades of research, response pathways, mechanisms, and regulators have been revealed and extensively studied. While most of this work has described such mechanisms in model plants and algae, expanding our view into new organisms, especially from extreme environments, bears great unrealized potential. Photosynthetic members of the desert biological sand crust (hereafter BSC) consortium must maintain positive net primary productivity through some of the harshest conditions on Earth in order to survive (Ohad *et al*., 2010; Fig. 1a,b). Among them are frequent hydration–dehydration cycles, a vast surface temperature amplitude from freezing along winter nights up to 60°C in mid‐summer afternoons, and extreme illumination levels (2000 μmol photons m^−2^ s^−1^, hereafter EIL; Supporting Information Fig. S1; Büdel & Veste, 2008; Couradeau *et al*., 2016). Due to the frequent use of the term *high light* to describe a wide and context‐dependent range of illuminations, we refer here to EIL to distinguish light intensities that are several‐fold above those required to saturate CO_2_‐dependent O_2_ evolution in a given organism, usually corresponding to 2000 μmol photons m^−2^ s^−1^ or above. During the last few decades, desert BSC algae and cyanobacteria have been the subject of in‐depth organismal studies (Murik *et al*., 2017; Oren *et al*., 2019; Oren *et al*., 2021). Among them, the green alga *Chlorella ohadii* (originally referred to as a contaminant of *Microcoleus* sp. and designated McX; Treves *et al*., 2013; Kedem *et al*., 2021b; Fig. 1c,d) stood out for being an attractive highly resistant and robust biological model system (Dann & Leister, 2017; Cardona *et al*., 2018; Kleine *et al*., 2021; Hitchcock *et al*., 2022; Leister, 2023), while exhibiting elevated photosynthetic and growth rates. Phylogenetic analysis has confirmed that *C. ohadii* is a *Chlorella* sp. and indicated that it belongs to a *C. sorokiniana* clade (Murik *et al*., 2024). However, numerous evolutionary modifications have made *C. ohadii* morphologically and physiologically distinguishable from *C. sorokiniana*; these distinct traits justify its classification as a separate species (additional info is available in AlgaeBase and NotulaeAlgarum). As we bring forward in this review, crucial for its survival is the ability to sense diurnal changes in environmental conditions and rapidly activate metabolism and growth in the short periods when water is available coincidentally with sufficient light. Here, we will present the main mechanistic insights accumulated in recent years on what allows *C. ohadii* to withstand extreme abiotic stress, mostly focusing on its resistance to EIL, which has been the subject of numerous physiological, spectroscopic, structural, and system biology‐directed studies (Fig. 2).

**Fig. 1:**
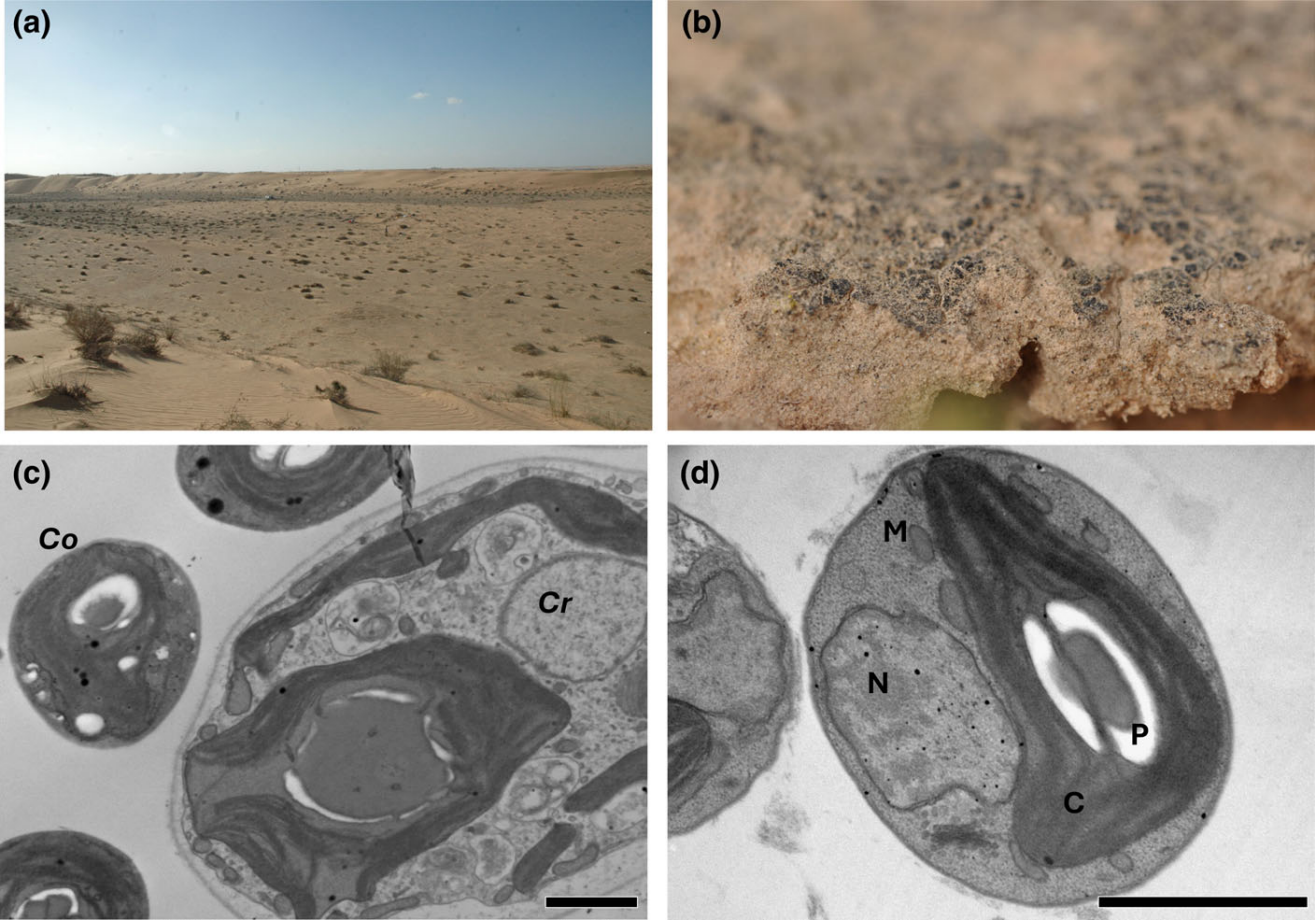
*Chlorella ohadii* was isolated from harsh desert sand crusts. Biological sand crusts on Nizzana station in the NW Negev, Israel (30°56′25″N; 34°22′55″E) in wide (a) and closeup (b) views. (b) Note the dark pigmentation associated with the filamentous cyanobacteria. Images were captured by Haim Treves. (c, d) TEM images (extracted from Treves *et al*., 2013) showing *C. ohadii* (*Co*) next to *C. reinhardtii* (*Cr*) cells, illustrating the size difference (c) and a magnified image of *C. ohadii* (d). Bars, 1 μm.

**Fig. 2:**
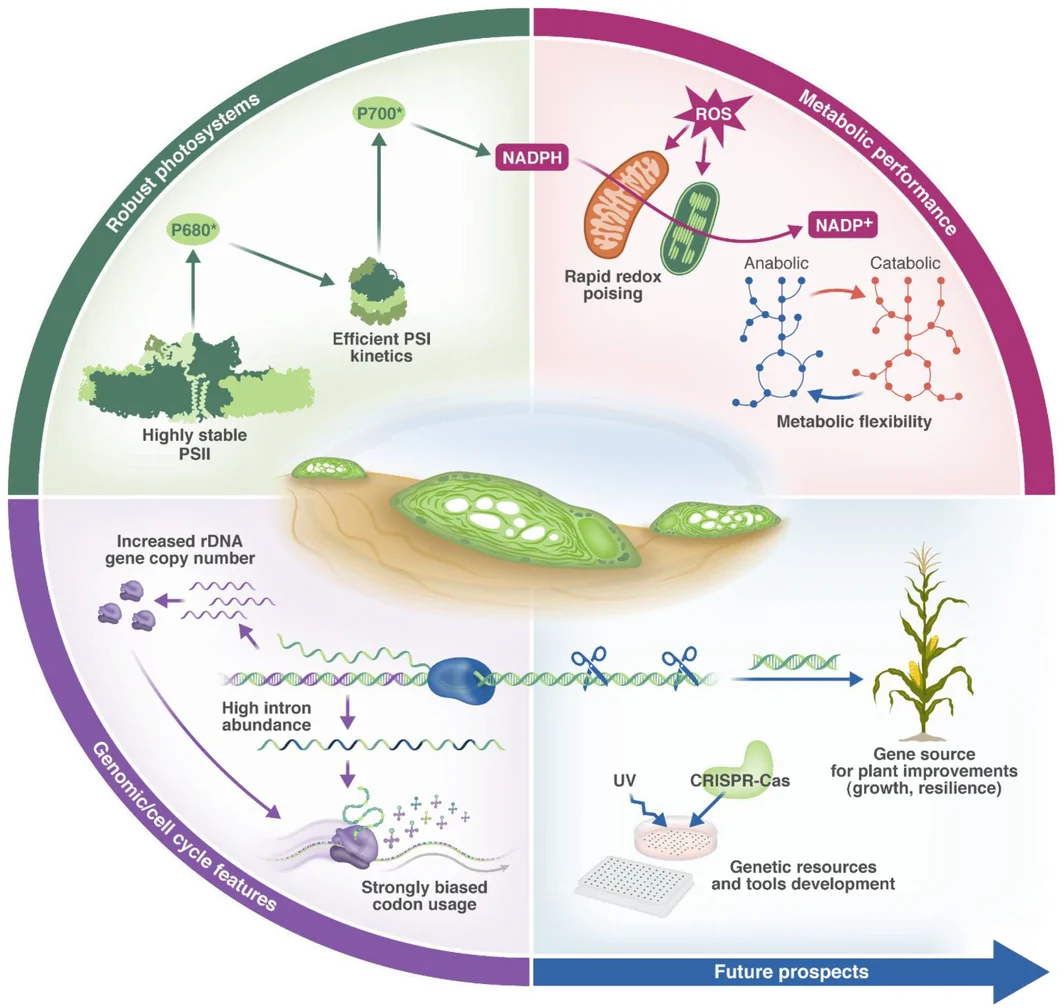
Multiple facets of *Chlorella ohadii* biological properties and potential as a research model and biotechnology platform. Illustration of the main concepts discussed in this review covering both *C. ohadii*'s extreme resistance and rapid growth features, relying on its biophysical (green), biochemical (magenta), and genomic (purple) properties.

## The mechanisms supporting *C. ohadii* resistance to EIL

Photosynthetic organisms experience damage to their photosystems, and particularly photosystem II (PSII), which is the more sensitive of the two photosystems, when exposed to illumination levels higher than those required to saturate CO_2_ fixation, through a process termed photoinhibition. The mechanisms leading to PSII photodamage and their regulation have been intensively studied and reviewed (Murata *et al*., 2007; Tyystjärvi, 2008; Erickson *et al*., 2015; Li *et al*., 2018; Roach *et al*., 2020 and references therein). Strikingly, *C. ohadii* is not only exceptionally resistant to photodamage under the conditions tested, but also exhibits elevated photosynthetic and growth performance under EIL (Treves *et al*., 2016). In this section, we will recapitulate the evidence accumulated on the numerous biophysical, biochemical, and metabolic adaptations underlying this resistance.

### Highly stable PSII

An intriguing feature of *C. ohadii* response and tolerance of extreme illumination draws on the robustness of its photosystem reaction centers, and especially PSII. First, several reports point to an altered functioning of its PSII based on rapid electron transfer kinetics, supporting higher stability rather than solely faster or more efficient repair mechanisms, as also supported by the minimal ^1^O_2_ formation documented in this alga (Treves *et al*., 2016). Further, both spectroscopic and structural evidence point to a massive (≥ 90%) cyclic electron flow operating around the PSII reaction center (PSII‐CEF; Treves *et al*., 2016; Ananyev *et al*., 2017; Fadeeva *et al*., 2023; Steiner *et al*., 2025). While additional canonical energy dissipation mechanisms are likely to act in parallel, PSII‐CEF are of great importance for research due to the scarce knowledge on such mechanisms in existing green models, and their clear biotechnological potential (indeed, several projects aiming to exploit *C. ohadii* features (i.e. EIL resistance, rapid metabolic, and growth rates) have commenced in the last decade (Kleine *et al*., 2021; Hitchcock *et al*., 2022; Treves, 2023)). Upon a shift from low‐light (100 μmol photons m^−2^ s^−1^, LL) to EIL, immediate activation of PSII‐CEF has been shown to result in faster Q_A_ site reoxidation, while maintaining an identical quantum yield (the highest per Chl QY reported for any phototroph without exogenous electron acceptor addition – 256 μmol O_2_ mol^−1^ Chl*a* per flash; Ananyev *et al*., 2017). Comparatively, recovery of most linear electron flow was observed within minutes of application of darkness or LL (Treves *et al*., 2016; Ananyev *et al*., 2017; Zournas *et al*., 2023). Considering the sharp temporal abiotic gradients (light, humidity) dominating the crusts, such rapid acclimation rates may be crucial for its survival. Further, structural analyses provided evidence for tighter binding of *C. ohadii* Q_B_ to its hydrophobic binding pocket (Fadeeva *et al*., 2023; Arshad *et al*., 2026), indicating a direct electron transfer from Q_A_^−^ to Q_B_ and the fast transfer of electrons from Q_B_^−^ to the quinone pool, and likely underlying the protective PSII‐CEF mechanism. Such a mechanism is also in line with the lower sensitivity to DCMU (3‐(3,4‐dichlorophenyl)‐1,1‐dimethylurea, known to compete with quinone for the Q_B_‐binding pocket) demonstrated by *C. ohadii* cultures (Treves *et al*., 2016). Structural studies of *C. ohadii* identified several subunits that are frequently absent in other published PSII structures, likely due to their weak binding and subsequent loss during the isolation procedure (Fadeeva *et al*., 2023; Arshad *et al*., 2026). These include the subunits PsbO, PsbP, and PsbQ constituting the oxygen‐evolving complex, which form a stronger association with the core complex through less flexible interactions, leading to a more rigid conformation possibly contributing to higher stability of the *C. ohadii* core complex (Fadeeva *et al*., 2023; Arshad *et al*., 2026). A similar case was identified for PsbR, which forms in *C. ohadii* extensive interactions with several core complex subunits and also with PsbY (Fadeeva *et al*., 2023; Arshad *et al*., 2026). These interactions between the two subunits and between them and the PSII core complex are also likely to play a role in stabilizing this alga's PSII under harsh conditions.

The very small light‐harvesting antenna is another contributing factor to minimized photodamage in *C. ohadii*. LL‐grown *C. ohadii* cultures naturally posses an unusually small antenna and very high Chl*a* : Chl*b* ratio (*c*. 12) compared to any other algae, including marine, freshwater, and other *Chlorella* spp. (Nakamura *et al*., 1976; Wood, 1979; Kowallik & Schürmann, 1984), whereas EIL cultures have practically no PSII antenna (Treves *et al*., 2016; Ananyev *et al*., 2017; Levin *et al*., 2021, 2023). Manipulating antenna size may be a useful strategy to mitigate photodamage, as previously demonstrated in *C. reinhardtii*, where mutants exhibiting a Chl*a* : Chl*b* ratio of 5–6 exhibited higher photosynthetic rates under saturating light (Mitra *et al*., 2012; Negi *et al*., 2020). More recently, specific linker light‐harvesting proteins (Lhcb) have been identified in *C. ohadii*, modulating its LHCII antenna size through binding not only S‐, but also M‐ and L‐LHCII trimers to the core complex dimer (Arshad *et al*., 2026). Resolving Lhcb in *C. reinhardtii* PSII structures has so far been limited by their suspected heterogeneous composition and the partial association flexibility of the M‐ and L‐LHCII trimers. In the case of *C. ohadii*, two of the Lhcb occupy the peripheral side and three others are located in inner positions of the LHCII trimers. Two of the latter were downregulated alongside M‐ and L‐LHCII trimers under high‐light, resulting in a reduction in the number of bound LHCII trimers and providing an alternative photoprotective mechanism in the absence of LHCSR (Arshad *et al*., 2026). Notably, while antenna size distinguished LL‐ and EIL‐grown cells (Levin *et al*., 2025), no changes were detected in PSII effective cross‐section or in carotenoid content during a short‐term shift between LL and EIL (Treves *et al*., 2016). The latter, alongside with the absence of an LHCSR homolog in the *C. ohadii* genome (Murik *et al*., 2024) and the lack of apparent state transitions, precluded a major role for nonphotochemical‐quenching‐dependant processes (hereafter NPQ, reaching *c*. 0.2 in EIL‐grown cultures) in the photoprotection of *C. ohadii* (Treves *et al*., 2016; Levin *et al*., 2023, 2025). In this regard, the aquatic green alga *Botryosphaerella sudetica*, isolated from high‐light exposed floating biofilms, has recently been shown to operate constitutive accumulation of PsbS and a qE‐based NPQ mechanism (Blifernez‐Klassen *et al*., 2026). Together with the information provided above on *C. ohadii* and *C. reinhardtii*, this example demonstrates the diversity in the composition of NPQ mechanisms, their extent and regulation within green algae, and reflects species‐specific adaptations to their life history.

In this respect, it should be noted that *C. ohadii* possesses two PsbS variants (Treves *et al*., 2020). While a recent report detecting low levels of the PsbS protein in EIL‐grown cells (Levin *et al*., 2023) supported the minor role played by NPQ in *C. ohadii* photoprotection, early (15 min) transcriptional upregulation of the *psbS* gene during a shift from LL to EIL (Treves *et al*., 2020), and the relatively high abundance of PsbS protein under EIL (97^th^ percentile, Table S1), indicated that its role may have been overlooked. This discrepancy may result from low reactivity of the PsbS antibody with *C. ohadii's* variant, or differences in illumination conditions (e.g. light quality and penetration, temperature) applied to EIL‐treated cells between different setups (see the Perspectives and future guidelines section). Further, detailed investigation of PsbS peptides from MS analysis reveals that one of the *C. ohadii* PsbS variants is neither expressed under LL nor EIL conditions, while the other accumulates massively under EIL, with *c*. 50‐fold increase compared with LL (Table S1), highlighting that its role in *C. ohadii* remains unclear, especially compared to its well‐established function in NPQ. In this context, a recent study in *C. reinhardtii* implicated PsbS in acclimation to HL beyond the mere activation of qE, affecting antenna organization, morphology and electron transfer at the PSI acceptor side, thus providing a possible explanation for its expression patterns under EIL in *C. ohadii* despite a minor role played by NPQ (Redekop *et al*., 2020).

A similar case was reported for the plastid terminal oxidase (PTOX), which was highly abundant in proteome analyses (Table S1), and has been found to play a role in EIL‐grown autotrophic cultures based on chlororespiration levels measured via membrane inlet mass spectrometry with stable O_2_ isotopes (Kedem *et al*., 2021a), but only found in low levels in other reports (Levin *et al*., 2023). An additional potential protein involved in photoprotection in *C. ohadii* is the LHC‐like carotene biosynthesis‐related protein (CBR), which has been reported to accumulate during growth under EIL (Levin *et al*., 2023). Sequence and carotenoid‐binding features of CBRs raised the hypothesis that this protein may sense luminal pH and respond by binding carotenoids and chlorophylls from degraded LHCs to facilitate energy dissipation from excited chlorophylls. Finally, EIL‐induced stacking of *C. ohadii* thylakoid membranes (Treves *et al*., 2016), alongside its very small antenna, proposed a role for a spillover‐like effect between its PSII reaction centers (Treves *et al*., 2020) and between them and its highly efficient PSI discussed in the next sections, gaining recent support from studies of wet–dry transitions in this alga (Kawamura *et al*., 2026).

### Faster PSI energy transfer kinetics

*Chlorella ohadii* adaptation to EIL should also be manifested in the structure and function of its PSI. Indeed, structural analysis of the PSI complex from EIL‐grown *C. ohadii* cells revealed the absence of several subunits known to bind LHCII under state transition II, including the core‐complex subunit PsaO (Caspy *et al*., 2021), consistent with the lack of apparent state‐transition induction (Levin *et al*., 2024). In some PSI populations under EIL, both PsaH and part of light‐harvesting antenna complex I (LHCI) were also missing from the structure (Caspy *et al*., 2021). Interestingly, the phosphoproteome of *C. ohadii* PSI revealed differential phosphorylation of several LHCI subunits, which was most pronounced in Lhca7 and Lhca6; the latter was also reported to undergo phosphorylation in *C. reinhardtii* (Younas *et al*., 2023), but occurred also in PsaO and Lhca1, for which no previous reports of phosphorylation existed in land plants or algae (Levin *et al*., 2024). This coincided with the presence of PsaO in the *C. ohadii* PSI complex in LL, but not EIL (Caspy *et al*., 2021). The observations that (1) many of the phosphorylation sites were localized on the interface of two subunits, and (2) growth light intensity affected the phosphorylation of LHC1 proteins and the elimination of PsaO, which is hypothesized to minimize PSI photodamage, give rise to the possibility that light‐mediated phosphorylation plays a role in stabilizing the different structural forms of PSI‐LHCI reported in LL and EIL. Another potential consequence of these structural features of *C. ohadii* PSI complexes is altered kinetics of energy transfer to its PSI reaction centers observed under LL, containing slower components which are absent in either *C. reinhardtii* or the cyanobacterium *Synechocystis* sp. PCC 6803 (Cherepanov *et al*., 2024). However, whether and to what extent this pattern of energy transfer kinetics is retained under EIL remains to be elucidated. Exploiting *C. ohadii* PSI may enhance the development of artificial photobiocatalytic systems, where one of the main concerns is the stability of photo‐bioelectrochemical cells (Yehezkeli *et al*., 2014). Thanks to higher stability and faster kinetics, *C. ohadii* PSI may offer a promising approach to bridge this gap by direct integration into such cells or inspiring design of artificial components based on a better understanding of its structure–function relationship (Terasaki *et al*., 2009; Yehezkeli *et al*., 2012).

Further, light‐modulated structural changes in *C. ohadii* PSI were accompanied by altered pigment composition of LHCI between EIL‐ and LL‐grown cells, with about half of the Chl*b* molecules replaced by Chl*a* in EIL, supporting faster electron transfer rates via higher excitation efficiency between pigment molecules (Caspy *et al*., 2021). The higher efficiency of PSI under EIL may also act more efficiently to alleviate PSII overreduction under these conditions, which have also been shown to induce stacking of thylakoid membranes in *C. ohadii*. Such stacking results in smaller distances between PSII reaction centers and between them and PSI reaction centers, increasing the probability of excitation energy transfer from PSII reaction centers and providing a possible quenching mechanism (Treves *et al*., 2016, 2020).

Consistent with decreased PSII antenna in EIL‐grown cells, an increase in PSI‐CEF has been observed (Levin *et al*., 2025), poising ATP : NADPH production ratio and possibly contributing to the increased ATP demand of faster growth, protein synthesis (Treves *et al*., 2022a), and CO_2_ concentration mechanism (CCM) activation (Treves *et al*., 2016, 2020) observed in *C. ohadii* under these conditions. This decrease was accompanied by a relatively small (1.4‐fold) increase in PGR5 (Levin *et al*., 2025) or even a decrease in EIL vs LL proteomes (Table S1), suggesting that, quantitatively, the observed increase in PSII‐CEF (Treves *et al*., 2016; Ananyev *et al*., 2017; Fadeeva *et al*., 2023; Steiner *et al*., 2025) had a larger effect via the PSI donor side. Differences in PGR5 abundance may also represent an example for variance between experimental setups (see the Perspectives and future guidelines section). Notably, PSI oxidation is slower in EIL vs LL‐grown cells measured in the absence of PSII inhibition, despite slower PSI reduction due to decreased PSII antenna and PSII‐CEF, indicating massive PSI‐CEF activation (see fig. 6 in Levin *et al*., 2025). This significant increase in PSI‐CEF is even more pronounced in light of smaller PSI acceptor side limitation consequent on increased metabolic NADPH oxidation in EIL cells, further discussed in the following section.

## *C. ohadii* metabolic performance under EIL

Crucial for survival under this wide range of illumination levels is the capacity to harness the consequent rates of ATP and reducing power accumulation into metabolism and growth, while detoxifying excess harmful byproducts. This is especially challenging in photosynthetic cells where the balance between anabolic and catabolic reactions must be tightly regulated to avoid futile cycles (Stitt *et al*., 2021). To address these partially contradicting demands, *C. ohadii* had to develop several adaptations during its evolution in the desert crusts, which are manifested in its metabolic capacities and their unique regulatory mechanisms, including rapid redox signaling and increased sink capacity, as detailed below.

One example, and a recurring motif in *C. ohadii* physiology, is the maintenance of relatively high basal metabolic capacities evident across different key pathways. This is likely an adaptive consequence of the unusual diurnal cycle in the desert crusts, allowing only a very short daily period of sufficient humidity once sunlight becomes available (Treves *et al*., 2020). The mere fact that photosynthesis and growth rates more than doubled promptly following a 30‐fold increase in incident light indicated that *C. ohadii* is a high‐light‐ready cell (Treves *et al*., 2020). This has been shown to strongly rely on elevated metabolic sink capacity, allowing rapid increases of over 10‐fold and 3‐fold in cellular starch and lipid content, respectively (Treves *et al*., 2016, 2020).

An analogous trend was observed when comparing chloroplast O_2_ consumption rates of *C. ohadii* cultures grown under LL and EIL. These rates reflect the sum of several processes, including PTOX, the Mehler reaction, and oxygenase activity of Rubisco. LL‐grown cultures of *C. ohadii* exhibited 1.5‐ to 2‐fold higher chloroplast O_2_ consumption rates, regardless of medium CO_2_ levels, which, together with the use of inhibitors, indicated that most of this difference is driven by high electron flux via PTOX under LL (Kedem *et al*., 2021a). In addition, PTOX was found at similar levels in both LL and EIL (Table S1; Levin *et al*., 2025). Thus, PTOX provides an additional constitutive valve allowing *C. ohadii* to withstand overreduction until a new metabolic balance (i.e. increased fluxes via the Calvin–Benson cycle (CBC) and to end‐products) under EIL is established (Treves *et al*., 2022a; Küken *et al*., 2023).

In this respect, noteworthy is *C. ohadii*'s redox poising capacity, evident in the altered reduction state of *c*. 85% of its redox‐responsive proteome within 3 min of shift to EIL, resulting in elevated and even overshooting levels of NADPH oxidation shortly after (Treves *et al*., 2020). Such an increase could also be detected, though to a lesser extent, when comparing EIL‐ to LL‐grown cultures (Kedem *et al*., 2021a). The altered *in vivo* redox response identified in several enzymes mediating export of reducing equivalents from the chloroplast and excess NADPH utilization (i.e. NADP‐MDH and NADP‐GAPDH; Ocheretina *et al*., 1993; Lemaire *et al*., 2005; Treves *et al*., 2020) conforms with a rapid redox balance switch, allowing a more oxidized NADPH system within minutes of shift to EIL (Fig. 3). At the same time, no redox response could be detected during EIL shift in the CBC redox regulator protein CP12 (Treves *et al*., 2020), whose levels were decreased in EIL‐ vs LL‐grown cultures (Table S1) despite much higher carbon fluxes via the CBC in the former (Treves *et al*., 2022a). These observations indicated that while this protein is involved in the upregulation of CBC flux upon transition from darkness (Marri *et al*., 2009), its role in EIL acclimation may be limited.

**Fig. 3:**
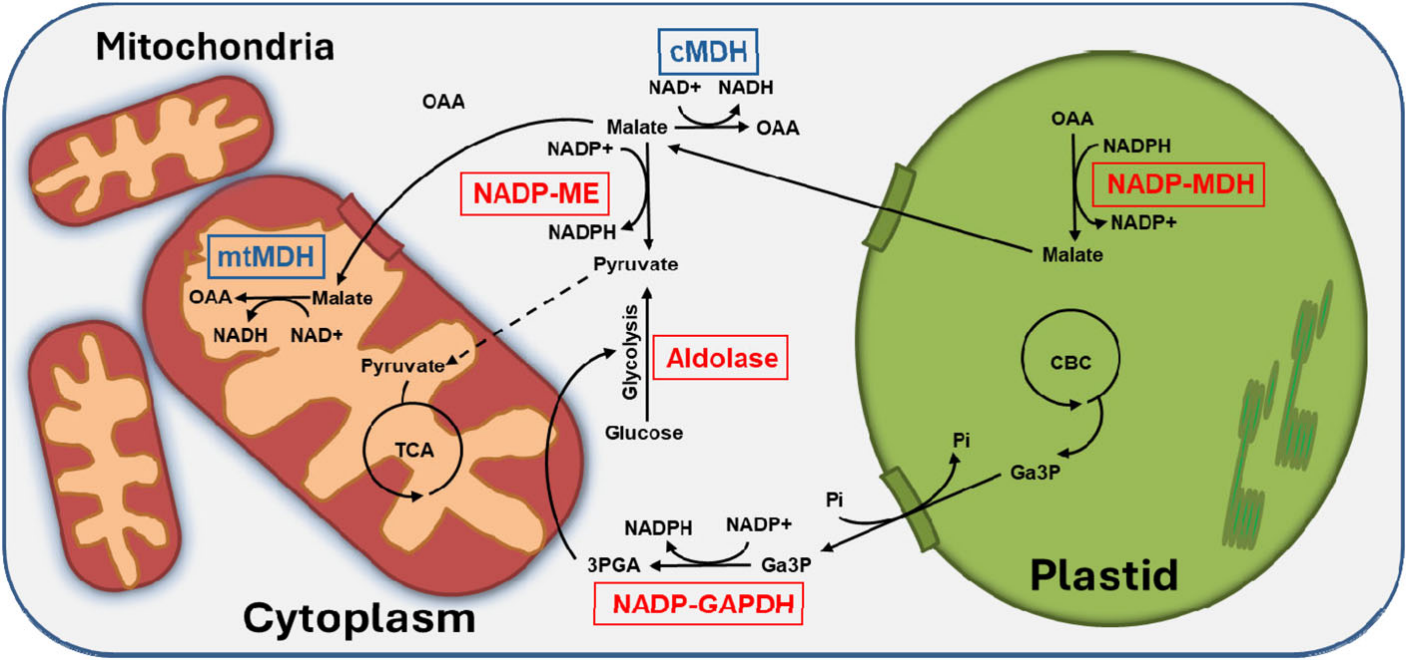
Cellular scheme of NADPH oxidation‐associated redox response of *Chlorella ohadii* (reconstructed from Treves *et al*., 2020). New redox balance characterized by lower cellular NADPH levels is rapidly reestablished following shift to EIL through the concerted operation of several components of NADPH oxidation pathways. Plastid NADP‐dependent malic dehydrogenase (NADP‐MDH) and cytosolic aldolase lacking key regulatory cysteines are not subject to post‐translational redox regulation, allowing higher basal activity. This coincides with the rapid redox response of the cytosolic nonphosphorylating NADP‐dependent glyceraldehyde‐3‐phosphate dehydrogenase (NADP‐GAPDH) and NADP‐dependent malic enzyme (NADP‐ME), involved in excess redox shuttling from the plastid and its downstream oxidation. Notably, NADP‐ME has only been previously found to be redox‐regulated in C_4_ plants.

The robustness of *C. ohadii* photosystems ensures a continuous elevated supply of ATP and NADPH for carbon fixation upon EIL exposure, inducing carbon limitation rather than photodamage, as reflected by upregulation of all photorespiratory genes within 15 min (Treves *et al*., 2020; Fig. S2). Consequently, during a shift from LL to EIL, transcriptional upregulation of many CCM‐associated genes has been observed, with apparent affinity for inorganic carbon (K_1/2_(Ci)) in *C. ohadii* reaching as low as 10 μM (Treves *et al*., 2016; Murik *et al*., 2024), comparable with highly efficient cyanobacterial CCMs measured under very low Ci (Sültemeyer *et al*., 1995; Mackenzie *et al*., 2004). It seems, however, that prolonged exposure to EIL compromises this efficiency, possibly due to impairment of the starch sheath surrounding the pyrenoid, also manifested by higher photorespiratory flux (Treves *et al*., 2020; Treves *et al*., 2022a; Fig. S2). Further, while many transcriptionally EIL‐upregulated genes were known CCM regulators and components (e.g. LCIB, HLA3, and CCP1), several uncharacterized proteins were regulated by either EIL or low CO_2_ (Treves *et al*., 2020), indicating new regulators may arise from exploring CCM in *Chlorella*. In this context, noteworthy is the ability of linker proteins from *C. sorokinana* mediating pyrenoid development (CSlinker) to induce the formation of Rubisco condensates *in planta* across various taxa (Barrett *et al*., 2024). The significant similarity between CSlinker and its *C. ohadii* homolog, alongside its attractivity as a plant‐compatible anchor for Rubisco matrix (Barrett *et al*., 2026), highlights the potential of studying structural components of *C. ohadii* CCM for pyrenoid engineering in plants.

Lastly, maintaining the metabolic sink capacity required for supporting such rapid EIL acclimation and ATP production must be accompanied by active sensing and neutralization of toxic metabolic intermediates (Huang *et al*., 2016). Indeed, contrary to reports from *C. reinhardtii*, H_2_O_2_ detoxification (ascorbate peroxidase (APX) activity) has been found to be constitutive rather than being induced by excess light or H_2_O_2_ pretreatment (Murik *et al*., 2014; Treves *et al*., 2016, 2020). This is in line with the similar expression levels detected for major reactive oxygen species (ROS) metabolism enzymes in the proteomes of LL‐ and EIL‐grown cultures, including superoxide dismutase (SOD), glutathione peroxidase (GPx), and APX (Table S1). At least one variant of APX and GPx was even downregulated in EIL, pointing to decreased ROS production under these conditions via CBC activation (Treves *et al*., 2022a), increased NADPH oxidation (Fig. 3; Treves *et al*., 2020), or both. This was also demonstrated in the lower rates of ^1^O_2_ production measured in isolated chloroplasts from LL‐ vs EIL‐grown cells (Levin *et al*., 2025). However, despite this difference, H_2_O_2_ accumulated to similar amounts under both conditions, providing additional evidence for the constitutive levels of ROS detoxification described above even under LL.

In summary, harboring both robust photosystems and highly efficient downstream redox metabolism sets *C. ohadii* as highly adapted to EIL conditions and enables rapid acclimation to LL‐EIL shift. It remains unclear to what extent *C. ohadii* metabolic capacity, especially for NADPH oxidation, laid the foundation for the development of its highly stable photosystems, or whether the latter constituted the driving force for its efficient redox utilization. While future studies on the evolution of photosynthesis‐related traits across the *Chlorella* clade may shed light on these questions, conservation of unique sequence patterns (e.g. absence of highly conserved cysteines) in key metabolic enzymes between *C. ohadii* and other *Chlorella* spp. (Treves *et al*., 2020), together with higher EIL sensitivity of its closest relative, *C. sorokiniana* (Treves *et al*., 2016; Murik *et al*., 2024), hints on metabolic capacities preceding the development of a highly resistant PSII in *C. ohadii* as a more likely scenario. The relationship between *C. ohadii* metabolic features and growth is discussed in the next section.

## The metabolic features underlying *C. ohadii*'s rapid growth rate

To the best of our knowledge, *C. ohadii* grows faster than any other eukaryotic photosynthetic organism, and remarkably exhibits growth rates similar to the maximum attained by unicellular nonphotosynthetic eukaryotes, even when grown photoautotrophically. Comparable rates in phototrophs have so far only been described in rapidly growing unicellular cyanobacteria (J. Yu *et al*., 2015). Operating within generation time frames of *c*. 1.5–2.5 h (Treves *et al*., 2013, 2017; Ananyev *et al*., 2017), it is therefore not surprising that *C. ohadii* cultures demonstrate rapid acclimation rates, shifting to a new apparent Km for Ci following a shift from mixotrophic to autotrophic conditions (Treves *et al*., 2016), and reestablishing new steady state photosynthetic rates upon transfer from LL to EIL within 15–20 min (Treves *et al*., 2020). Further, photosynthetic Vmax rates as high as 600–700 μmol O_2_ mg^−1^ Chl h^−1^ were recorded under several conditions (Treves *et al*., 2013, 2016, 2020), more than double that of *C. reinhardtii* (Virtanen *et al*., 2021; Treves *et al*., 2022a) or *Phaeodactylum tricornutum* (Onno Feikema *et al*., 2006) and comparable with maximal rates documented in cyanobacteria (Thomas *et al*., 1993). As already outlined, such rates may represent an adaptive strategy to the limited temporal window available for photosynthesis and growth in the desert BSC.

In recent studies, it has become clear that, similar to its roles in EIL acclimation, *C. ohadii* metabolism is also a key factor in enabling its fast growth. When compared to other green algae (*C. reinhardtii*, *C. sorokiniana*), and C_3_ (Arabidopsis) and C_4_ (maize) plant models, several differential metabolic flux patterns were identified extending beyond the higher metabolic fluxes via the CBC expected from its higher photosynthetic rates (Geffen *et al*., 2023; Küken *et al*., 2023). ^13^C‐Labeling kinetics revealed substantial flow of fixed C from PEP into the TCA cycle in *C. ohadii*, imperative for replacing TCA cycle intermediates consumed by the biosynthetic pathways promoting investment into growth (e.g. amino acid biosynthesis). Indeed, empirical protein synthesis rates measured in *C. ohadii*, *C. reinhardtii*, and *C. sorokiniana* correlated tightly with growth (Treves *et al*., 2022a). This increased demand for anaplerotic fluxes via PEP carboxylase provides a plausible explanation for the coupling found between CBC and lower glycolysis labeling in these algae compared with land plants, especially pronounced in *C. ohadii*. The faster flux to RuBP in algae found in Treves *et al*. (2022a) has been hypothesized to draw on the metabolic requirements of algal CCM (Treves *et al*., 2022b). Noteworthy is also the substantial flux of fixed C found in sucrose in *C. ohadii*, which was undetectable in *C. reinhardtii* (Treves *et al*., 2020), in line with the role played by this hydroxyl‐rich sugar as an osmoprotectant in the former (Kedem *et al*., 2021a). In this respect, desiccation tolerance of *C. ohadii* has been shown to depend on co‐culture with crust cyanobacteria, or on a slower dehydration rate combined with external provision of trehalose or sucrose (Kedem *et al*., 2021a). As sucrose is the main exported sugar and photosynthetic end product in land plants, this allows using *C. ohadii* as a green algal system to study its synthesis in photosynthetic cells. Moreover, while per weight levels of most primary C metabolism intermediates were comparable between *C. ohadii*, Arabidopsis, and maize, their corresponding levels in *C. reinhardtii* were 2‐ to 10‐fold lower in most cases (Szecowka *et al*., 2013; Arrivault *et al*., 2017; Treves *et al*., 2022a). Aiming to point to specific reactions underlying its faster growth and the observed differential flux patterns identified, a compartmentalized genome‐scale metabolic model (GEM) has been recently constructed for *C. ohadii*. This model predicted several reactions involved in fluxes not only into the TCA cycle and PEP but also into the oxidative pentose phosphate pathway, distinguishing *C. ohadii* from slower‐growing algae, including the reactions catalyzed by 6‐phosphogluconolactonase (Pgl) and pyruvate phosphate dikinase (PPDK; Soleymani *et al*., 2025). Among these differential patterns, targeting the route of C from the CBC to PEP for improvement in plants using insights gained from *C. ohadii* is deemed attractive due to PEP's roles in plant growth and development (Prabhakar *et al*., 2010) and potential limitations of its synthesis (Ma *et al*., 2014; Polle *et al*., 2014).

The elevated metabolic rates reported above are to be expected considering such high photosynthetic and growth rates. However, other metabolic features also seem to contribute to *C. ohadii* growth properties and have likely been shaped during its adaptation to the desert environment. Particularly, *C. ohadii* exhibits a multiphasic growth pattern, which has been shown via metabolic profiling to be driven by metabolic shifts between photosynthate accumulation and utilization (Treves *et al*., 2017), likely reflecting imprinting of the desert diurnal cycle synchronizing the cultures (Treves *et al*., 2020). This metabolic flexibility presumably facilitates *in situ* growth optimization through boosting photosynthesis during early morning, and growth and respiration upon rewetting from night dew formation (Ohad *et al*., 2010). Indeed, inhibition of this multiphasic pattern resulted in limited growth even when cultures are provided with higher levels of organic carbon source (Treves *et al*., 2017). Cellular levels of polyamines have been implicated in regulating these shifts (and affecting *C. ohadii* respiration rates), as also supported by modulation of carbon fluxes into polyamine biosynthesis in response to changing illumination levels (Treves *et al*., 2017, 2022a; Lavandosque & Vischi Winck, 2025). However, this response may also be related to polyamine accumulation in the thylakoid membrane under EIL, which is suggested to play a role in photoprotection (Arshad *et al*., 2026). The combination of this metabolic flexibility with high metabolic rates also inspired studies aiming to exploit *C. ohadii* in wastewater bioremediation, demonstrating its potential and revealing higher rates of removal of both metals and key nutrients (N, P, and C) compared to commonly used microalgae (Subramaniam, 2024).

## The genomic features associated with fast growth

Efficient function of *C. ohadii* transcriptional and translational machineries is a prerequisite for keeping the short cell cycles concomitant with its logarithmic growth phases (Treves *et al*., 2017, 2020). *Chlorella ohadii* genomic arrangement and characteristics have evolved accordingly to harbor a high copy number of ribosome‐encoding genes (occupying most of its transcriptome's top expressed genes) alongside increased codon bias compared to other green algal genomes (Murik *et al*., 2024). The latter is thought to allow faster translation and, hence, protein synthesis and growth (C.‐H. Yu *et al*., 2015). An intriguing feature of the *C. ohadii* genome entails harboring elevated per‐gene intron abundance (12.01) compared with other chlorophyte algae, with introns occupying as much as a quarter of its genome size at the expense of intergenic space (Treves *et al*., 2020; Murik *et al*., 2024). Interestingly, despite this massive expansion of intron content, levels of alternative splice variants in its transcriptome were comparable to those of other models (Treves *et al*., 2020; Murik *et al*., 2024). This observation, alongside the apparent positive correlation between intron abundance and growth rate across the *Chlorella* clade, gave rise to the hypothesis that increasing intron abundance may serve as an adaptation conferring increased genomic stability, in view of the lower mutation rates identified in genes with higher intron content (Monroe *et al*., 2022). Whether and which of these mechanisms act alone or in concert to support *C. ohadii*'s exceptional growth, and whether the observed relationship between intron abundance, growth rate, and transcript abundance in this alga is merely a random correlation remain a major research avenue in deciphering *C. ohadii* biology once genetic tools become available for this alga.

## Increasing need for genetic tools targeting *C. ohadii*

Several aspects render *C. ohadii* ideal as a potential green model for photosynthesis research. In addition to several recently developed resources including a draft genome (Murik *et al*., 2024), full metabolic model (Soleymani *et al*., 2025), and established procedures for omics research (Treves *et al*., 2020, 2022a; Geffen *et al*., 2023; Levin *et al*., 2024, 2025), this alga carries inherent features which are simply not found in other photosynthetic systems. These include yeast‐level doubling times (up to 1.5–2 h), very high photosynthetic and metabolic rates (five‐ and threefold higher C assimilation rates compared with *C. reinhardtii* or Arabidopsis, respectively; Treves *et al*., 2022a) and wide resource media utilization flexibility. Further, as already outlined above, several aspects of its biology make it a highly relevant and favorable model for studying plant physiology, photosynthesis, and metabolism. However, amidst several attempts, no stable transgene expression has been established in *C. ohadii* to date, imposing the main gap for turning this alga into a widely used model. Transformation of *C. ohadii* is complicated by several factors, including its rigid cell wall, a wide antibiotic resistance repertoire, and efficient silencing of foreign genes. For further reading on potential approaches to overcome these limitations, see Notes S1 and Fig. S3.

## Perspectives and future guidelines

The fascinating case of *C. ohadii* has further emphasized the immense potential of exploring life in extremes, especially in photosynthetic organisms, with the underlying notion being that desert organisms must have developed unique adaptations to cope with prevailing harsh conditions. As such, *C. ohadii* is currently investigated as a gene resource for improved photosynthesis and resilience in crop plants (Fig. 2). In one approach, researchers aim to import key subunits supporting its photosystems' robustness into plants (Dann & Leister, 2017) with recent structural insights and new predictions tools (Zabret *et al*., 2021) facilitating the development of chimeric designs carrying potentially improved compatibility. Capitalizing on its dynamic metabolism and fast flow rates through photosynthesis and primary carbon metabolism, another approach aims to export metabolic enzymes, regulators, or architectures from *C. ohadii* to be implemented in model algae and plants for increased biomass accumulation rates (Treves *et al*., 2022a; Treves, 2023). Further, very high respiration rates (up to anoxia) recorded in *C. ohadii* (Treves *et al*., 2017), consequent on its rapid growth, set this alga as a promising platform for hydrogen overaccumulation (Kedem *et al*., 2021a) once genetic tools become available. Given the unusual diurnal cycle in the desert favoring fast‐growing photoautotrophs and the extreme illumination levels driving elevated NADPH oxidation and respiration rates, it is intriguing to speculate how many additional highly performing algae can be identified through exploring this and similar habitats (Malavasi *et al*., 2020; Box 1).

Box 1 **The search for new photosynthesis**

Textbook photosynthesis as we know it mostly takes place under controlled environments where oxygen levels, humidity, and carbon availability alter within a limited range. By contrast, photosynthetic microbes found in harsh arid conditions often face dramatic changes that forcibly change the arena (Raanan *et al*., 2016; Treves *et al*., 2016). Hence, the extreme radiation in such ecological habitats often results in surplus electron flow and is accompanied by the generation of excess ROS and protein damage (Ohad *et al*., 2010; Raanan *et al*., 2016; Treves *et al*., 2020). However, a less studied feature of such habitats is hypoxia or anoxia developing in certain windows of the day. Not only that oxygen in dense, microbe‐populated niches may be scarce, but the necessity to use an extremely short window for growth during sunrise or sunset may also be accompanied by ultrafast respiration, which further decreases its availability (Treves *et al*., 2017, 2020). Such a surrounding environment, together with tight functional and morphological coordination between the plastid and mitochondria (Elman *et al*., 2022; Dhawan & Rao, 2025), forces oxygenic photosynthesis to be carried out under semi to full anoxic conditions (Fig. 4a). These changes have dramatic implications on the ability of oxygen to serve as the main electron acceptor for surplus electrons via major pathways like the Mehler reaction (Kozuleva *et al*., 2021), PTOX and flavodiiron proteins (Flvs) routes (Liran *et al*., 2016). The absence of such crucial valves forces the activation of alternative mechanisms. These include an anoxic switch slowing down photosystem II activity (Fig. 4b; Milrad *et al*., 2023); diverting surplus electrons to hydrogenase for hydrogen production (Milrad *et al*., 2021, 2023) and further, yet to be discovered, mechanisms. This anoxic switch, which is widely spread in desert isolates (Yacoby, unpublished results), diminishes PSII activity by threefold within seconds of illumination, and was suggested to involve restriction of electron transfer at the PSII acceptor side, possibly via intrinsic changes in its structure altering its electron output. This may prevent overexcitation of the photosynthetic apparatus, thereby mitigating oxidative damage. Hence, isolating new species from harsh environments and studying their response to extreme illuminations, and especially photosynthesis under anoxic conditions, can provide us with novel mechanistic knowledge and uncover exotic key regulators of oxygenic photosynthesis.

**Fig. 4:**
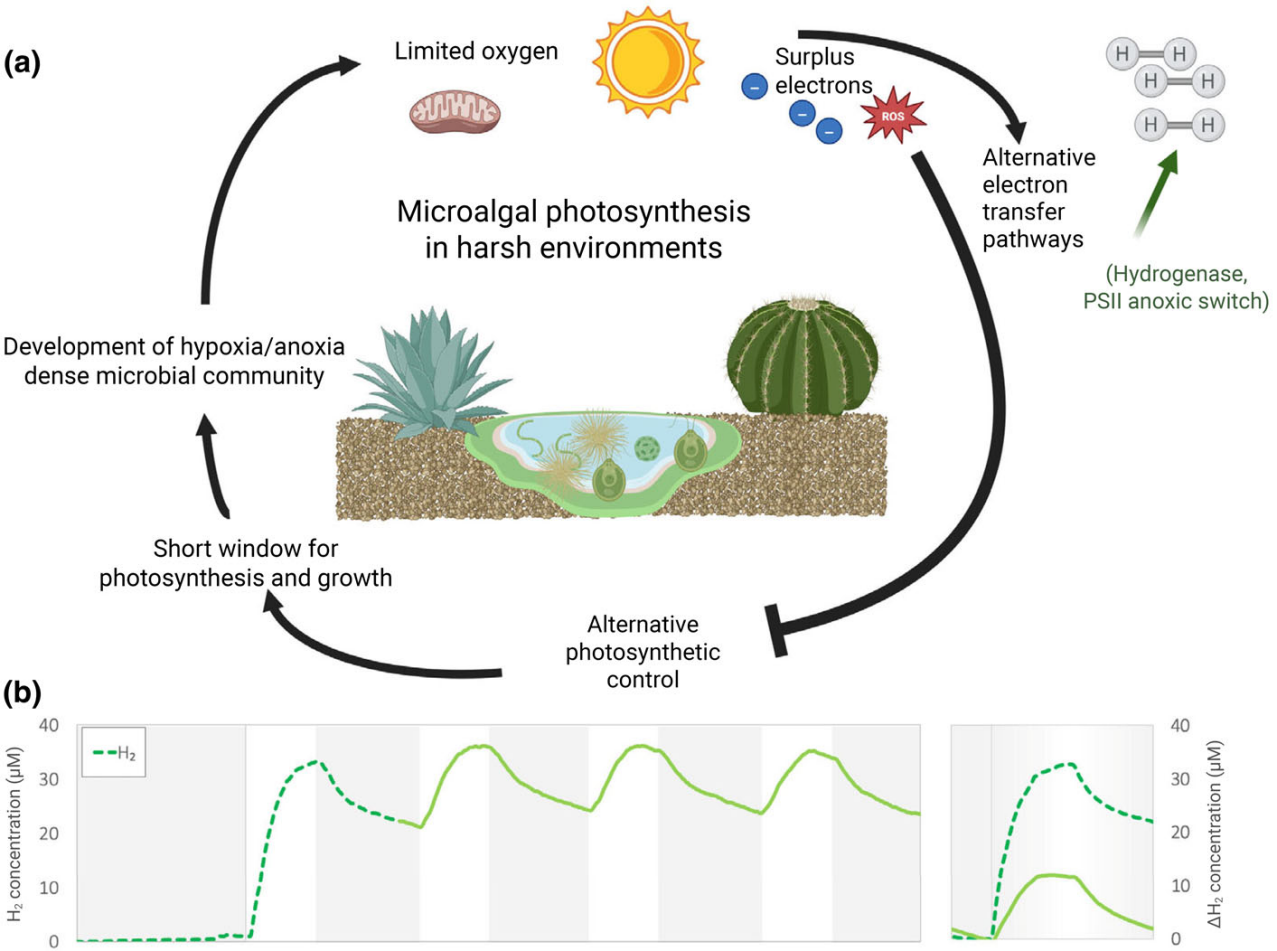
Adaptive photosynthetic control and the PSII anoxic switch in arid microalgae. (a) Schematic illustration of the regulatory cycle of microalgal photosynthesis in harsh, oxygen‐limited environments where dense microbial communities lead to the rapid development of hypoxia and anoxia. In these conditions, initial light exposure generates surplus electrons, enhancing reactive oxygen species (ROS) accumulation. To mitigate this, cells employ alternative electron transfer pathways, for example, via hydrogenase. The PSII anoxic switch represents a new level of alternative photosynthetic control that acts as a regulatory brake. (b) As shown in the hydrogen concentration graphs, while initial light exposure (dashed green line) results in a rapid H_2_ burst, subsequent light exposures (solid green line) exhibit significantly slower hydrogen production. These measurements serve as a diagnostic tool, informing us about the anoxic switch activation and the extent to which it actively downregulates photosynthetic rates to manage the short window available for photosynthesis and growth while preventing oxidative damage.

Consequently, as much of this work involves studying nonmodel organisms, which are in many cases the research subject of fewer groups, it is imperative to establish community standards and guidelines ensuring reproducibility and comparability between studies. Several examples can be found in the case study of *C. ohadii*. Specifically, when investigating the effects of EIL, sufficient light penetration must be controlled for and short light‐path experimental setups and/or low‐density cultures are to be favored. At the very least, reporting of incident light during growth and treatments should include applied and in‐culture levels. In addition, as most tools are developed and optimized for existing model systems (e.g. *C. reinhardtii*), studies should be designed and adapted to test the effects of different stressors under optimal or natural growth conditions for a given nonmodel organism, in order to dissect the response components and regulators associated with a certain trait within its relevant eco‐physiological context, for example, temperature range. The latter is further underlined by the role of metabolism in abiotic stress responses, and the tight relationship between temperature and enzyme activity, but should be extended to other abiotic factors (osmotic conditions, nutrient availability, etc.).

## Outstanding questions in *C. ohadii* biology research

- What are the main transgene silencing mechanisms operating in *C. ohadii*, and how can those be manipulated to allow stable genetic transformation of this alga?
- How did *C. ohadii* mitochondrial function adapt to keep pace with its elevated growth and photosynthetic rates? Which cellular and molecular factors play a role in supporting its respiration and NADPH oxidation rates?
- What are the evolutionary forces driving *C. ohadii*'s increased intron abundance, and which molecular mechanisms associate introns with growth and gene expression in *C. ohadii* cells?
- What enables *C. ohadii* to cope with additional abiotic stressors in the harsh desert crusts, including the wide temperature amplitude and osmotic range? In addition, what role is played by light perception and specific photoreceptors in preparation for stress (as demonstrated for BSC cyanobacteria; Oren *et al*., 2019; Xu *et al*., 2025; Bai *et al*., 2026)?
- How is *C. ohadii* CCM regulated? What are the master transcriptional regulators, and how are they distinguished from well‐studied algae, like *C. reinhardtii*?
- Given the unusual diurnal and seasonal cycles in the desert BSC, what are the features of the *C. ohadii* life cycle? Is sexual reproduction playing a role thereof? And if yes, what is the relative balance of sexual vs asexual reproduction phases?

## Competing interests

None declared.

## Author contributions

HT and MB conceived the framework. OG, MB, IY and HT prepared the initial draft. OG, HM and HT created the figures. HT integrated the sections and finalized the manuscript. All authors read and approved the final version of the manuscript.

## Disclaimer

The New Phytologist Foundation remains neutral with regard to jurisdictional claims in maps and in any institutional affiliations.

## Supporting information


**Fig. S1** Meteorological parameter development during a summer day in Nizzana station.
**Fig. S2** Multi‐omics illustration of *Chlorella ohadii* responses to EIL.
**Fig. S3** Design principle of an automated UV mutagenesis and high‐density phenotyping platform for *Chlorella ohadii*.
**Notes S1** Challenges and current state in the development of genetic tools targeting *Chlorella ohadii*.
**Table S1** MS abundance data of proteins discussed in this review.
Please note: Wiley is not responsible for the content or functionality of any Supporting Information supplied by the authors. Any queries (other than missing material) should be directed to the *New Phytologist* Central Office.
