# Supplementary material for: Extending the limits of algal growth and stress resilience – the desert alga Chlorella ohadii as an emerging model
Source: New Phytol. 2026 Oct 1;252(5):1787–98. doi: 10.1111/nph.71587 (PMC13738669; doi:10.1111/nph.71587)
Supplement: Supplementary file 1 [file NPH-252-1787-s001.docx]

## *New Phytologist* Supporting Information

Article title: Extending the limits of algal growth and stress resilience - the desert alga *Chlorella ohadii* as an emerging model

Authors: Or Geffen, Hilla Matityahu, Iftach Yacoby, Michal Breker, Haim Treves

Article acceptance date: 31 August 2026

The following Supporting Information is available for this article:

**Fig. S1** **Meteorological parameters development during a summer day in Nizzana station.** Progress of temperature at 50 cm above surface (orange), relative humidity (green) and radiation (blue) along a summer day in the Nizzana desert station where *C. ohadii* was isolated from. Data are available from <https://www.meteo-tech.co.il/>. Early morning dew is the main source of humidity for about 200 nights per year. Evaporation of dew water due to a rapid increase in air temperature and declining humidity results in severe desiccation shortly after sunrise. Temperature directly at surface reaches 60°C during mid-summer afternoons (Büdel and Veste 2008).

**Fig. S2 Multi-omics illustration of *C. ohadii* responses of to EIL.** Multi-omics illustration of *C. ohadii* temporal responses to EIL treatments associated with CCM, photorespiration and starch synthesis extracted from Treves et al, 2020. Genes, proteins, and metabolites presented showed significantly higher (red) or lower (blue) levels of expression, redox-response, and accumulation, respectively. Arrows illustrate potential positive (red), and negative (blue), direct (solid line), and indirect (dashed line) effects, according to the model suggested in the text on CCM response to EIL.

**Fig. S3 Design principle of an automated UV-mutagenesis and high-density phenotyping platform for *C. ohadii*.** Schematic illustration of a potential genetic screening pipeline for *C. ohadii*. The workflow integrates UV mutagenesis of wild-type cells, automated colony picking, large-scale arraying of mutant libraries, and parallel phenotyping through biomass accumulation and growth-curve profiling. Downstream stages include microscopy-based trait characterization, genetic and metabolic analyses, and data integration for systems-level interpretation.

**Table S1 MS abundance data of proteins discussed in this review.** Fold changes in protein abundance (LFQ) between photoautotrophic cultures grown under EIL and LL (n=3 biologically independent experiments, Benjamini-Hochberg corrected P (FDR) are presented). Percentile EIL and LL represents the protein abundances based on their iBAQ values compared with the entire proteomes under EIL or LL.

| Gene | Description | Fold change EIL/LL | FDR | Percentile LL | Percentile EIL |
| --- | --- | --- | --- | --- | --- |
| g1958 | SOD | 1.215099211 | 0.03753 | 99.7 | 99.9 |
| g5099 | SOD2 | 0.237912089 | 0.024366 | 75 | 44 |
| g10134 | GPX | 0.221578874 | 0.00726 | 96 | 90 |
| g9383 | GPX2 | 0 | N/A | 0 | 0 |
| g10267 | APX | 0.462565998 | 9.59E-05 | 86 | 83 |
| g7865 | APX2 | 0.57020898 | 0.011763 | 73 | 72 |
| g5696 | PTOX | 0.865991185 | 0.788397 | 63 | 52 |
| g7724 | PsbS | 47.30523662 | 0.00832 | 34 | 97 |
| g7725 | PsbS2 | 0 | NA | 0 | 0 |
| g4850 | CP12 | 0.294859507 | 0.007344721 | 99.6 | 99.2 |
| g911 | GPR5 | 0.098657449 | 0.001442 | 97.2 | 84.4 |
| g3699 | HYDA1 | 0 | NA | 0 | 0 |
| g554 | HYDA2 | 0.65307628 | 0.33 | 43.4 | 54.1 |

**Notes S1** **Challenges and current state in development of genetic tools targeting *C. ohadii*** During our attempts to introduce a selection marker of resistance to G418, one of the few antibiotic agents allowing effective selection in *C. ohadii*, no verification of stable expression could be obtained from immunoblots. These observations, together with the loss of resistant colonies only several transfers following transformation, suggested highly active silencing mechanism is in action. Until future work will address these challenges in *C. ohadii* molecular biology, additional alternative approaches for developing genetic resources for this alga are necessary. We have recently optimized an automated large-scale UV mutagenesis pipeline tailored for *C. ohadii* to overcome the current lack of stable transgene expression (Figure S3), based on our prior experience in establishing this pipeline in *C. reinhardtii* (Breker *et al.*, 2016; Breker *et al.*, 2018). Ongoing efforts now focus on expanding this platform toward a genome-wide mutant library, combining optimized UV mutagenesis with multiplexed sequencing and improved genome assembly to facilitate accurate identification of causal variants. This approach permits continuous monitoring of population-wide and single-mutant growth dynamics under a range of environmental regimes. Testing the growth performance of these colonies under diverse light intensities, nutrient availabilities, and stress conditions will lay the foundations for large-scale forward-genetic and quantitative genotype-phenotype studies in *C. ohadii.* Such tools offer a powerful leverage in establishing genetic transformation of this alga, as previously demonstrated for *C. reinhardtii* through the isolation of UV mutants impaired with transgene silencing (e.g. UVM4, UVM11) which became widely used as background strains (Neupert *et al.*, 2009; Neupert *et al.*, 2020). Together with improvements in genome annotation and the establishment of reliable transformation and transient expression systems, these resources are expected to form the basis for stable genetic manipulation in *C. ohadii*, ultimately transforming this exceptional alga from a physiological model into a fully tractable experimental system for studying photosynthesis and stress resilience.

**References**

**Breker M, Lieberman K, Cross FR. 2018.** Comprehensive Discovery of Cell-Cycle-Essential Pathways in Chlamydomonas reinhardtii. *Plant Cell* **30**(6): 1178-1198.

**Breker M, Lieberman K, Tulin F, Cross FR. 2016.** High-Throughput Robotically Assisted Isolation of Temperature-sensitive Lethal Mutants in Chlamydomonas reinhardtii. *J Vis Exp*(118).

**Neupert J, Gallaher SD, Lu Y, Strenkert D, Segal Na, Barahimipour R, Fitz-Gibbon ST, Schroda M, Merchant SS, Bock R. 2020.** An epigenetic gene silencing pathway selectively acting on transgenic DNA in the green alga Chlamydomonas. *Nature Communications* **11**(1): 6269.

**Neupert J, Karcher D, Bock R. 2009.** Generation of Chlamydomonas strains that efficiently express nuclear transgenes. *The Plant Journal* **57**(6): 1140-1150.
